# Prevalence of postpartum post-traumatic stress disorder and associated factors among postnatal mothers in West Arsi zone, South West Ethiopia, 2024: a community-based cross-sectional study

**DOI:** 10.3389/fpsyt.2024.1470819

**Published:** 2024-10-28

**Authors:** Solomon Seyife Alemu, Mohammedamin Hajure Jarso, Negeso Gebeyehu Gejo, Habtemu Jarso Hebo, Daniel Yohannes Bedecha, Firomsa Bekele, Wubishet Gezimu, Addisalem Workie Demsash, Sheleme Mengistu Teferi, Gemeda Wakgari Kitil, Geleta Nenko Dube, Awol Arega Yimer, Berhanu Negese Kebede, Gemechu Gelan Bekele, Lema Fikadu Wedajo

**Affiliations:** ^1^ Department of Midwifery, College of Health Science, Madda Walabu University, Shashemene, Ethiopia; ^2^ Department of Psychiatry, College of Health Science, Madda Walabu University, Shashemene, Ethiopia; ^3^ Department of Public Health, College of Health Science, Madda Walabu University, Shashemene, Ethiopia; ^4^ Department of Pharmacy, Institute of Health Science, Wallaga University, Nekemte, Ethiopia; ^5^ Department of Nursing, College of Health Science, Mattu University, Mattu, Ethiopia; ^6^ Department of Health Informatics, Asrat Woldeyes Health Science Campus, Debre Berhan University, Debrebirhan, Ethiopia; ^7^ Department of Midwifery, College of Health Science, Madda Walabu University, Bale Goba, Ethiopia; ^8^ Department of Midwifery, College of Health Science, Mattu University, Mattu, Ethiopia; ^9^ Department of Health Informatics, College of Health Science, Mattu University, Mattu, Ethiopia; ^10^ Department of Midwifery, College of Medicine and Health Sciences, Arba Minch University, Arba Minch, Ethiopia; ^11^ Department of Midwifery, Institute of Health Science, Wallaga University, Nekemte, Ethiopia

**Keywords:** posttraumatic stress disorder, postpartum, mothers, West Arsi, Ethiopia

## Abstract

**Background:**

Up to one-third of women globally experience giving birth as traumatic, which can lead to postpartum post-traumatic stress disorder. Postpartum post-traumatic stress disorders have significant health consequences for the mother, child, and other family members. Although it has tragic health impacts, little is known about this problem in the study area.

**Objectives:**

The study aimed to assess the prevalence of postpartum posttraumatic stress disorder and associated factors among postnatal mothers.

**Methods:**

A community-based cross-sectional study was employed among 635 mothers in the first year after childbirth by using simple random sampling techniques from March 20 to April 20, 2024 in West Arsi zone, Ethiopia. Face-to-face interviewers administered structured questionnaires that were used to collect the data. The collected data were cleaned, coded, and entered into Epidata and exported to Statistical Package for Social Sciences for further analysis. Both bivariate and multivariable analyses were done by using binary logistic regression.

**Result:**

Out of 635, 624 postnatal mothers participated in the study, for a response rate of 98.27%. The prevalence of postpartum post-traumatic stress disorder was 21.60% (95% CI: 18.40%, 24.87%). Primiparous mothers (AOR = 2.26, 95% CI: 1.38, 3.70), have no antenatal care follow-up (AOR = 2.48, 95% CI: 1.47, 4.20), cesarean section delivery (AOR = 2.86, 95% CI: 1.50, 5.61), instrumental delivery (AOR = 3.06, 95% CI: 1.75, 5.34), maternal morbidity (AOR = 2.94, 95% CI: 1.71, 5.05), and postpartum intimate partner violence (AOR = 7.43, 95% CI: 4.53, 12.20) were the identified factors.

**Conclusion and recommendation:**

As identified, one out of five mothers had postpartum posttraumatic stress disorder. Thus, healthcare providers should focus on identified factors like cesarean section and instrumental deliveries while counseling, as this enhances the mothers’ psychological readiness. In addition, the West Arsi Zonal Health Office should develop effective strategies to alleviate the problem by focusing on the identified factors.

## Introduction

Childbirth is a milestone event in the lives of reproductive-age women and marks the beginning of motherhood. It is an intense event that is accompanied by extreme physical as well as psychosocial stress (1, 2). Stress is defined as a reaction to a change in the environment, which may be adaptive or non-adaptive. Post-traumatic stress disorder (PTSD) is a condition marked by an inability to recuperate following exposure to or observation of a traumatic event. Postpartum post-traumatic stress disorder (PPTSD) is among the problems typically occurring after childbirth in the context of a traumatic birth and other unpleasant childbirth-related events (3).

It is characterized by a symptomatic triad of re-experiencing (flashbacks and nightmares), avoidance (staying away from reminders), and arousal (reactive sweating and palpitations). It typically presents a few weeks to several months after exposure to an exceptionally shocking, threatening, or catastrophic event in the absence of an organic cause (4).

Postpartum PTSD, which possesses unique attributes, antecedents, and outcomes, is a disorder arising after a traumatic birth experience, leading to specific negative maternal symptoms and poor mother–infant outcomes (5). It is diagnosed 4 weeks onwards after the initial traumatic event; onset can also be delayed at 6 months or more after the event (6). The trauma experienced by postpartum women could be a negative perception of the birthing process (7).

Childbirth can be a traumatic event for the mother due to objective and subjective stressors such as obstetric complications in labor and delivery and an emergency cesarean section as well as the woman’s negative appraisal of the birth (8). Up to two out of three women perceive having suffered obstetric violence during childbirth (9). Furthermore, during childbirth, women may experience obstetric violence, like incorrect or inappropriate treatment, either physically or emotionally, with inadequate clinical care or with a violation of the principle of autonomy (10).

The National Institute for Health and Care Excellence (NICE) defined traumatic birth as a criterion “A” qualifying event for PTSD in women: “Traumatic birth includes births, whether preterm or full term, which are physically traumatic (for example, instrumental or assisted deliveries or emergency cesarean sections, severe perianal tears, postpartum hemorrhage) and births that are experienced as traumatic, even when the delivery is obstetrically straightforward” (11). Thus, in the wake of a traumatic childbirth experience, postpartum women suffer from a posttraumatic stress disorder (8).

It is a distressing condition that women with postpartum PTSD are less likely to seek out medical care for themselves or their babies, are less likely to breastfeed, have much more difficulty with attachment and bonding, are less likely to have another child, or are trying to control the process via an epidural or a cesarean section, even when not medically indicated. They are also facing challenges in their relationships, both emotionally and physically (7).

Globally, the prevalence of postpartum PTSD in the community sample was estimated to be between 3.1% and 24.5% (12–14). The prevalence of postpartum PTSD is a significant health problem for both high- and low-income countries. It varies and is estimated from 3.4% to 20.7% in developed countries (15–21) and from 3.6% to 28.57% in low-income countries (22–24).

The maternal postpartum mental health problem has a wide range of impacts, particularly if it is not known and left untreated (18). Mental health conditions including PPTSD account for 1 in 5 years of life lived with disability globally, leading to more than US$ 1 trillion per year in economic losses, early mortality of 10–20 years, and a high number of suicide deaths (close to 800,000 deaths per year). Perinatal PTSD symptoms also result in adverse perinatal (mother–infant) outcomes (25) including maternal depression, prematurity, and low birth weight (5).

Postpartum PTSD has an adverse impact on children’s social–emotional development, and it has effects on parenting stress (26). Moreover, 26% of the children had a moderate risk of developmental delay, and 9% had a high risk of developmental delay following maternal postpartum stress disorder (27). Evidence showed that women who had postpartum PTSD suffered fear of childbirth in the subsequent pregnancy, which affects their experience of pregnancy and, in turn, can affect fetal development (28). It was implied that identifying and treating P-PTSD is very important for maternal and neonatal health (29).

The problem was found to be affected by several factors, like low partner support, lack of private health insurance, unplanned pregnancy, pressure to have an induction and epidural analgesia, emergency cesarean birth, not exclusively breastfeeding, depression, infant complications from traumatic previous births, and medical complications (21).

Pregnancy and childbirth with complications are potentially stressful situations since these are challenging events that often have a long-term emotional sequel and can predispose mothers to PPTSD (30, 31). Conditions like perinatal trauma (specifically perinatal complications and abuse) were identified as among the antecedents of PPTSD (5). Additionally, mistreatment during obstetric care, physical and verbal abuse, and discrimination are also among the factors that increased the prevalence of posttraumatic PTSD (32).

A systematic review and meta-analysis in five sub-Saharan African countries reported that the prevalence of disrespect and abuse during maternity care was 15%–98% (10). As revealed from the study conducted in West Arsi zone, Shashemene town public health facilities, 37% of women getting maternity care reported that they have faced disrespect and abuse (violation of their human rights) that may push them to PPTSD (33).

The importance of screening, diagnosis, and management of perinatal mental health (PMH) conditions in maternal and child health (MCH) services has been highlighted in WHO recommendations on maternal and newborn care for a positive postnatal experience (34). Reports on maternal mental health in Africa suggested that maternal mental health must be prioritized to achieve the United Nations Sustainable Development Goals by 2030 and that the African region must rethink its approach to maternal mental health to provide a blueprint for future public health emergencies (35).

As indicated by studies, postpartum posttraumatic stress disorder is increasing and is relatively common, especially in the postpartum period, equivalent to the prevalence data for postpartum depression and anxiety; however, postpartum PTSD is underdiagnosed and undertreated (7). The problem has numerous impacts and is increasing, but it is neglected by obstetricians, clinicians, psychiatrists, and researchers in developing countries (36). Even though PPTSD has a significant impact on maternal mental health, infant health, and family member health, a little is known in Ethiopia. Therefore, this study aimed to investigate the prevalence of PPTSD and associated factors in West Arsi zone, Ethiopia, in 2024.

## Methods

### Study area and period

The study was conducted in West Arsi zone, which is found in the Oromia Regional State of Ethiopia. It covers an area of 11,776.72 km^2^. The zone lies between 6012′29″ and 7042′55 latitude and 38004′04″ and 39046′08″ longitude. The zone has 13 districts, namely, Shashemenne, Kokosa, Arsi Negele, Gadab Hasasa, Shalla, Wondo, Dodola, Adaba, Siraro, Kofale, Heban Arsi, Kore, and Nansabo, and two urban administrative districts, Arsi Negele and Kofale. Based on the estimated population in 2022, the zone has a total population of 2,929,894, of whom 13.85% are urban residents (37). Data was collected from March 20 to April 20, 2024.

### Study design

A community-based cross sectional study design was employed.

### Population

#### Source population

The study included all postnatal mothers who were in the first year after childbirth in West Arsi zone.

#### Study population

All postnatal mothers in the first year after childbirth in the selected districts in West Arsi zone during the data collection period were included.

### Eligibility criteria

A postpartum woman who resides in West Arsi zone and is in her first year following childbirth was included in this study, whereas mothers who were in their first 4 weeks after giving birth, mothers having PTSD diagnoses prior to having a baby, and mothers who were critically ill and unable to communicate were excluded.

### Sample size determination

Sample size was determined using single population proportion formula by considering the following assumptions: the proportion (*p*) of 50%, *Z* value at 95% confidence level (Zα/2) = 1.96, and 5% degree of precision. Substituting the above-mentioned assumptions in the formula, the minimum required sample size was calculated as 384. Adding 10% non-response rate and 1.5 design effect, the final sample size was 635.


$$n=\frac{Z^{2}*P(1-P)}{d^{2}}$$

where

*n* = the required sample size.

*z* = the value of the standard normal curve score corresponding to the given confidence level = 1.96.

*p* = proportion of population which is 50%.

*d* = permissible margin of error which is 5%.

### Sampling technique and procedure

The study was conducted among postpartum mothers found in West Arsi zone. According to the zonal health bureau information, West Arsi has a total of 13 districts. By taking the recommendation of WHO on sampling techniques, 30% of the zonal districts were included in the study. Thus, four districts, namely, Heban Arsi, Shashemenne, Gadab Hasasa, and Wondo districts, and their 18 kebeles were selected by the lottery method: Shashemenne district—from 19 kebeles, six kebeles were selected; Gadab Hasasa—from 20 kebeles, six kebeles were selected; Wondo—from eight kebeles, two kebeles were selected; and Heban Arsi—from 12 kebeles, four kebeles were selected. The total number of postpartum mothers found in the selected kebeles was 1,610 as per the report obtained from each district health office.

A list of postpartum women and the codes of households was obtained from the health extension workers’ family folder of each respective kebele, which was used as a sampling frame. The calculated sample size was proportionally allocated to each selected kebele proportional to their population size. Then, study samples were selected by computer-generated simple random sampling techniques from the sample frame. The selected mothers were interviewed at their homes. Health extension workers were used as guides during the home-to-home interview. In the case of a selected household with more than one eligible participant, only one participant was selected by lottery.

### Study variables

#### Dependent variable

Postpartum posttraumatic stress disorder is the dependent variable in this study.

#### Independent variables

Listed below are the independent variables in this study:

Socio-demographic variables—age, marital status, educational status, residence, religion, ethnicity, occupation, and household wealth index.

Obstetric and service-related variables—duration of postpartum period, parity, status of this pregnancy, history of abortion, ANC, frequency of ANC, maternal morbidity [direct maternal morbidity (obstetric hemorrhage, hypertensive disorders, obstructed labor, puerperal sepsis, gestational diabetes mellitus, and perianal tear) and indirect maternal morbidities (anemia, malaria, hypertension, asthma, tuberculosis, and HIV)], onset of labor, kind of labor onset, mode of delivery, nature of cesarean section, episiotomy, instrumental delivery, disrespect and obstetric violence, initiation of breastfeeding, and neonatal loss.

Psychosocial variables—social support, perceived safety during birth, and intimate partner violence.

### Operational definitions

#### Postpartum posttraumatic stress disorder

Those mothers who scored greater than or equal to 19 out of 56 were classified as having PPTSD and coded as “1” and those who scored less than 19 were classified as not having PPTSD and coded as “0” by using the Perinatal Post-traumatic Stress Disorder Questionnaire-II (38).

#### Social support

By using the Oslo Social Support Scale (OSSS-3), scores between 3 and 8 were classified as having poor social support and coded as “1”, scores of 9 to 12 were classified as having moderate social support and coded as “2”, and scores of 12 to 14 were classified as having strong social support and coded as “3” (39).

#### Intimate partner violence

This was assessed by using 13 items developed from an adapted tool to assess domestic violence against women in low-income country settings. These include physical violence, sexual violence, and psychological violence. Any mother who is a victim of at least one type of IPV was coded as “1”, and those who were not victims were coded as “0” (40).

Disrespect and abuse of women experienced during childbirth in a facility: Seven group item questionnaires with the specific categories were used to assess the disrespect and abuse of women experiencing during childbirth in facilities. Each category has “yes” or “no” dichotomized responses. A respondent was considered to have been disrespected and/or abused for the specific category if she reported “yes” to at least one of the verification criteria in that category and coded as “1” and if no yes to the items not disrespected and coded as “0” (42, 43).

Maternal morbidity: Any health condition attributed to and/or complicating pregnancy and childbirth that has a negative impact on the woman’s well-being and/or functioning (44).

Perceived safety during birth: The women were asked about the safety of birth (How do you perceive the safety of your recent childbirth)? and had responses as bad, fair, good, very good, and excellent (45).

Average monthly income: This was categorized as above poverty line and below poverty line based on the current classification system by the World Bank group as (US$1.90) or 97.85 Ethiopian Birr (ETB) daily or 2,935.5 ETB monthly. Monthly income that is below the poverty line was coded as “0” and that above the poverty line was coded as “1” (46).

Instrumental vaginal delivery: Those mothers who gave birth vaginally, accomplished with the aid of instruments, which can be vacuums or forceps (47).

### Data collection tool and procedure

A structured, interviewer-administered data collection tool was used for this study. The questionnaires have had questions about socio-demographic factors, obstetrics and health services, social support, and intimate partner violence and questions assessing PTSD symptoms. The questionnaires to assess socio-demographic status as well as obstetrics and health services-related factors were adapted from previous literatures. The WHO Multi-country Study on Women’s Health and Domestic Violence Against Women questionnaire was used to assess partner violence during the most recent pregnancy (41), and OSSS-3 (39) was used to assess social support.

The outcome variable, postpartum posttraumatic stress disorder, was assessed by using Perinatal Post-traumatic Stress Disorder Questionnaire-II. The tool has a 14-item measure assessing post-traumatic symptoms related to the childbirth experience, including intrusiveness or re-experiencing, avoidance behaviors, and hyper-arousal or numbing of responsiveness (48), with Cronbach’s alpha 0.92, and been validated in different countries. The questionnaire was prepared in English language and later translated into Afan Oromo for data collection. Six bachelor’s degree holder data collectors and four supervisors were recruited for data collection. Health extension workers were used as guides during the home-to-home interview. The data collectors explained the objectives of the study to be participated in by reading the consent sheet aloud in Afan Oromo language. Then, data was collected by face-to-face interview with the participants at home. Data collection was overseen by the supervisors on a daily basis.

### Data quality control

In order to assure the quality of the data, a pretest was done in Adaba district, which is found in West Arsi zone, on a sample of 64 (10% of the sample) mothers to check any ambiguities and difficulty. The internal consistency of the tool was checked, and it has Cronbach’s α test for the outcome variable PPTSD at 0.89. Initially, the tool was developed in the English language and translated to Afan Oromo for actual data collection, and the Afan Oromo version was retranslated back to English to cross-check the consistency of the tool. Training on the objectives of the study, data collectors, and participant safety for both data collectors and supervisors was given for 2 days. Supervision was carried out by the supervisors daily for the sake of clarity, accuracy, and consistency of the data.

### Data processing and analysis

Data was cleaned, coded, and entered into the Epidata version 3.1software. Then, it was exported to the Statistical Package for Social Sciences (SPSS) version 26 software for further analysis. Descriptive statistics such as mean, standard deviation, and percentage were determined. The association between the outcome variable, postpartum posttraumatic stress disorder, and each independent variable was seen in the binary logistic regression model.

A multi-co-linearity test was done using co-linearity statistics among the independent variables. In the second step, independent variables with a *p*-value <0.25 were retained and entered into the binary logistic regression model for multivariable analysis. The degree of association between the outcome and independent variables was determined using the OR with 95% CI and *p*-value. The model goodness of fit was tested by using Hosmer–Lemeshow test. A *p*-value <0.05 was considered as the cutoff point to declare statistically significant factors.

## Result

### Socio-demographic characteristics of the study participants

Out of 635 postpartum mothers, a total of 624 participated in the study, making the response rate 98.27%. The mean and standard deviation of the participants’ ages were 27.97 ± 5.23, and 93.1% of the study participants were married. As identified, 43.4% have received primary education, and 52.2% have an average monthly income below the poverty line. In addition, 66.5% of the study participants lived in rural residences (**Table 1**).

**Table 1:** Socio-demographic characteristics of postpartum mothers on postpartum posttraumatic stress disorder and associated factors in West Arsi zone, Ethiopia, 2024.

Variables	Categories	Frequency (624)	Percentage (100%)
Age of the study participants	18–24	170	27.2
	25–29	238	38.1
	30–34	91	14.6
	≥35	125	20.0
Marital status of the study participants	Single	40	6.4
	Married	581	93.1
	Widowed	3	0.5
Ethnicity of the study participants	Oromo	480	76.9
	Ahmara	86	13.8
	Tigre	40	6.4
	Others^a^	18	2.9
Religion of the study participants	Orthodox	239	38.3
	Muslim	342	54.8
	Protestant	37	5.9
	Others^b^	6	1.0
Educational level of the study participants	No formal education	67	10.7
	Primary (1–8)	271	43.4
	Secondary (9–12)	206	33.0
	Tertiary (diploma and above)	80	12.8
Occupation of the study participants	Housewife	345	55.3
	Merchants	135	21.6
	Government workers	125	20.0
	Others^c^	19	3.1
Average monthly income	Below poverty line	326	52.2
	Above poverty line	298	47.8
Residence of the study participants	Rural	415	66.5
	Urban	209	33.5

a Gurage, Sidama, and Wolayita.

b Wakeffata and Adventist.

c Students.

### Obstetric characteristics of the postpartum mothers

Out of the total number of study participants, 68.8% were multipara women, and 67.1% of the mothers had their condition planned during the index of pregnancy. In addition, 71.8% of the women have had at least one ANC follow-up with their recent baby, and 88.9% of them gave birth vaginally. As identified from this study, 65.4% of the postpartum women did not develop any complications during pregnancy, delivery, or the postpartum period. Similarly, 50.2% of the study participants were in their 3rd month and below of the postpartum period (**Table 2**).

**Table 2:** Obstetric characteristics of postpartum mothers on postpartum posttraumatic stress disorder and associated factors in West Arsi zone, Ethiopia, 2024.

Variables	Categories	Frequency	Percentage (100%)
Parity (624)	Primiparous	195	31.2
	Multiparous	429	68.8
Status of recent pregnancy (624)	Planned	419	67.1
	Not planned	205	32.9
History of abortion (624)	Yes	123	19.7
	No	501	80.3
ANC follow-up in recent baby (624)	Yes	448	71.8
	No	176	28.2
Number of ANC follow-up (448)	Once	198	44.2
	2 to 3 times	149	33.3
	≥4	101	22.5
Kind of labor onset in recent birth (624)	Spontaneous	560	89.7
	Induced	64	10.3
Mode of delivery in recent birth (624)	Cesarean	69	11.1
	Vaginally	555	88.9
Nature of cesarean (69)	Emergency cesarean delivery	59	87.0
	Elective cesarean delivery	10	13.0
Episiotomy during your recent delivery (624)	Yes	149	23.9
	No	475	76.1
Instrumental delivery during the recent delivery (624)	Yes	117	18.8
	No	507	81.2
Time of initiation of breastfeeding after delivery (624)	Within 1 h	357	57.8
	After 1 h	267	42.8
Maternal morbidity (624)	Yes	216	34.6
	No	408	65.4
History of neonatal loss	Yes	32	5.1
	No	592	94.9
Duration after recent delivery (in months) (624)	≤3 months	313	50.2
	4–6 months	243	38.9
	≥7 months	68	10.9

### Psychosocial interventions and health services-related characteristics of the postpartum mothers

This study identified that 43.9% of the postpartum mothers had moderate social support, and 34.3% of them faced disrespect and abuse during the recent childbirth. In addition, 32.2% of the postpartum mothers faced intimate partner violence during this postpartum period (**Table 3**).

**Table 3:** Psychosocial interventions and health services of postpartum mothers on postpartum posttraumatic stress disorder and associated factors in West Arsi zone, Ethiopia, 2024.

Variables	Categories	Frequency (624)	Percentage (100%)
Social support of study participants in recent childbirth	Poor	151	24.2
	Moderate	274	43.9
	Strong	199	31.9
Experience intense and distressing fear about the upcoming labor and childbirth	No fear/only slight apprehension	502	80.4
	Intensely distressing fear	122	19.6
Perceived birth safety of your recent pregnancy	Bad	73	11.7
	Fair	324	51.9
	Good	148	23.7
	Very good	15	2.4
	Excellent	64	10.3
Disrespect and abuse during recent childbirth	Faced disrespect and abuse	214	34.3
	Did not face disrespect and abuse	410	65.7
Intimate partner violence during postpartum period	Faced IPV	201	32.2
	Did not face IPV	423	67.8

### Prevalence of postpartum post-traumatic stress disorder among postpartum mothers in West Arsi zone, Ethiopia, 2024

**Figure 1:**
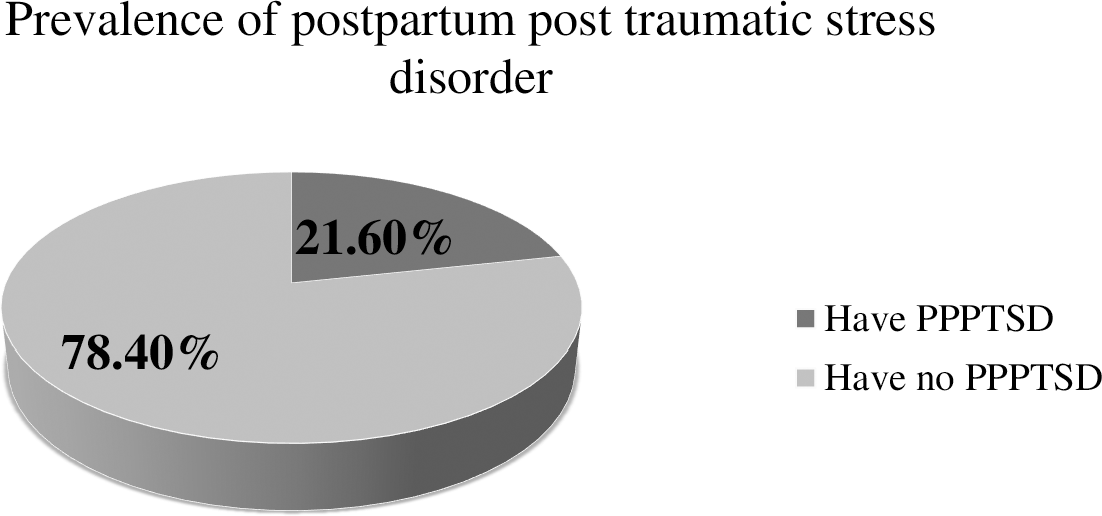
Diagrammatic representation of the sampling procedure for the study on prevalence of postpartum posttraumatic stress disorder in West Arsi zone, 2023/2024.

This study revealed that the prevalence of postpartum post-traumatic stress disorder among postpartum mothers was 21.60% (95% CI: 18.40%, 24.87%) (**Figure 1**).

### Factors associated with postpartum post-traumatic stress disorder among postpartum mothers in West Arsi zone, Ethiopia, 2024

In binary logistic regression, nine variables—parity, status of current pregnancy, ANC follow-up, mode of delivery, instrumental delivery, maternal morbidity, social support, disrespect and abuse during the recent childbirth, and postpartum intimate partner violence—were candidates for multivariable logistic regression. Finally, six variables like parity, ANC follow-up, mode of delivery, instrumental delivery, maternal morbidity, and intimate partner violence during the postpartum period fulfilled the criteria of association in the final model.

The study revealed that the odds of having postpartum post-traumatic stress disorder among primiparous mothers were 2.26 times higher compared to those of multiparous mothers (AOR = 2.26; 95% CI: 1.38, 3.70). In addition, the odds of having PPTSD among postnatal mothers who have had no ANC follow-up during their pregnancy were 2.48 times higher than their counterparts (AOR = 2.48; 95% CI: 1.47, 4.20).

Furthermore, the odds of postnatal mothers who gave birth by cesarean section to have postpartum traumatic stress disorders were 2.86 times more than those of postpartum mothers who gave birth vaginally (AOR = 2.86; 95% CI: 1.50, 5.61). Similarly, mothers who had an instrumental delivery during the recent childbirth had 3.06 times higher odds of developing postpartum posttraumatic stress disorder than mothers who did not (AOR = 3.06; 95% CI: 1.75, 5.34).

The odds of PPTSD among postnatal mothers who experienced maternal morbidity during their pregnancy, delivery, or postpartum period were 2.94 times higher than those who never experienced it (AOR = 2.94; 95% CI: 1.71, 5.05). Finally, the study identified that the odds of PPTSD among postnatal mothers who experienced postpartum intimate partner violence were 7.43 times higher than the odds of having the problem among their counterparts (AOR = 7.43; 95% CI: 4.53, 12.20) (**Table 4**).

**Table 4:** Binary and multivariable logistic regression on postpartum post-traumatic stress disorder among postpartum mothers in West Arsi zone, Ethiopia, 2024.

Variables	Categories	PPPTSD		COR (95% CI)	AOR (95% CI)
		Have PPPTSD (135)	Have no PPPTSD (489)		
Parity	Primipara	55 (28.2%)	140 (71.8%)	1.71 [1.15, 2.54]	2.26 [1.38, 3.70]^a^
	Multipara	80 (18.6%)	349 (81.4%)	1	1
Status of recent pregnancy	Planned	79 (18.9%)	340 (81.1%)	1	1
	Unplanned	56 (27.3%)	149 (72.7%)	1.62 [1.09, 2.40]	1.21 [0.70, 2.10]
ANC follow-up in recent baby	Yes	78 (17.4%)	370 (82.6%)	1	1
	No	57 (32.4%)	119 (67.6%)	2.27 [1.52, 3.39]	2.48 [1.47, 4.20]^a^
Mode of delivery	Cesarean	28 (40.6%)	41 (59.4%)	2.86 [1.69, 4.83]	2.86 [1.50, 5.61]^a^
	Vaginally	107 (19.3%)	448 (80.7%)	1	1
Instrumental delivery	Yes	43 (36.8%)	74 (63.2%)	2.62 [1.70, 4.10]	3.06 [1.75, 5.34]^a^
	No	92 (18.1%)	415 (81.9%)	1	1
Maternal morbidity	Yes	68 (31.5%)	148 (68.5%)	2.34 [1.59, 3.45]	2.94 [1.71, 5.05]^a^
	No	67 (16.4%)	341 (83.6%)	1	1
Social support of the study participants in recent childbirth	Poor	62 (41.1%)	89 (58.9%)	4.08 [2.45, 6.80]	1.92 [0.96, 3.84]
	Moderate	44 (16.1%)	230 (83.9%)	1.12 [0.64, 1.87]	1.23 [0.67, 2.26]
	Strong	29 (14.6%)	170 (85.4%)	1	1
Disrespect and abuse during recent childbirth	Faced disrespect and abuse	63 (29.4%)	151 (70.6%)	1.96 [2.45, 6.80]	1.30 [0.75, 2.27]
	Did not face disrespect and abuse	72 (17.6%)	338 (82.4%)	1	1
Intimate partner violence during postpartum period	Faced IPV	95 (47.3%)	106 (52.7%)	8.58 [5.60, 13.16]	7.43 [4.53, 12.20]^a^
	Did not faced IPV	40 (9.5%)	383 (90.5%)	1	1

1, reference group.

a *p*-value ≤0.05 in a multivariable analysis.

## Discussion

In recent times, evidence shows that several factors related to childbirth contribute to the occurrence of traumatic experiences that can result in posttraumatic stress disorder. This condition can have numerous detrimental effects on the lives of both the mother and her family members. Thus, this study points out the prevalence of postpartum posttraumatic stress disorder and associated factors among postpartum women in West Arsi zone, Ethiopia, in 2024. This study sought the prevalence of PPPTSD, which was 21.60%. As revealed by the study, parity, ANC follow-up, mode of delivery, instrumental delivery, maternal morbidity, and IPV were identified as significantly associated factors in the study setting.

As identified from our study, PPTSD in the study area was 21.60%. This is consistent with studies done in Germany with 21.17% (49), Switzerland with 20.10% (36), and England with 20.30% (50). This is supported by data from the literature, which indicates that childbirth is a potentially traumatic event that can lead to posttraumatic stress disorder (PTSD). Across the continent, 19.7% to 45.5% of women report that their childbirth was traumatic (51, 52). Consequently, this study demonstrates that the burden is present in low-income countries like Ethiopia and that the problem has not received enough attention in the past.

However, our finding is lower than the studies conducted in Morocco with 28.57% (48) and Switzerland with 63.90% (16). The possible discrepancies may be due to differences in sample size, as some mothers with the problem may be missed due to the small sample size, which can thus underestimate the magnitude of the problem. In addition, the difference in the study conducted in Switzerland may be due to the duration of the postnatal period, as a mother with less than 6 weeks of postnatal period was included. During this period, postpartum women are shown to exhibit traumatic symptoms, which may have interfered with their unpleasant labor and delivery-related fatigue and increased the severity of this problem.

However, the finding of this study is higher than those of studies conducted in Nigeria with 5.90% (53), China with 6.1% (22), Laka with 3.60% (24), Brazil with 9.80% (23), Israel with 3.40% (21), Australia with 5.70% (20), and Spain with 12.70% (9). This could be because of the varying socioeconomic status of the participants, the instruments used in each study, the sample size, the length of the postpartum period, and the length of the studies.

The study identified that parity was a factor significantly associated with PPPTSD. This is supported by a study conducted in Slovak women (54). This might be due to the fact that primiparous women are more likely to be linked to traumatic birth experiences, unexpected events during childbirth, and anxiety while giving birth. On the other hand, primiparous women may also experience more labor complications, like prolonged labor, genital tears, episiotomies, and others, than multiparous ones, which worsened the condition (55). In contrast to this finding, the study conducted in Brazil (23) revealed that multiparous women are more likely affected by PPTSD than primiparous women. This could be because multiparous women often have a history of trauma, past obstetric complications, and surgical delivery experiences.

This study identified that those mothers who have no ANC follow-up during the index of pregnancy are highly affected by posttraumatic stress disorder than who have ANC follow-up. This is supported by a study conducted in Switzerland (56). This might be due to the fact that during antenatal care follow-up, mothers may be aware of both physiological and psychological changes that may worsen the problem and can consult with health professionals to overcome the problem. Moreover, they get advice and counseling on birth preparedness, which may lessen the problem.

In addition, the variables that are found in this study are mode of delivery and instrumental delivery. PTSD is more common in mothers who had an instrumental delivery and a cesarean section than in mothers who gave birth vaginally and did not have an instrumental delivery. This is supported by studies conducted in Spain (17), Greece (57), and France (58). This is supported by the fact that the majority of women in low-income countries like Ethiopia, particularly those living in rural areas, feel bad or down about not giving birth naturally. They experienced pressure to give birth naturally, and they find it challenging to handle remarks made by others regarding instrumental and cesarean deliveries. Beyond this, complications associated with instrumental birth and cesarean delivery also contribute significantly to the development of common mental health issues like postpartum traumatic stress disorder (59).

Furthermore, this study also identified maternal morbidity as a significant factor. Mothers who had any maternal morbidity complications had a higher chance of being affected by PPTSD. This is supported by studies conducted in Brazil (60), Israel (21), and China (22). Furthermore, this is supported by a systematic review conducted on the relationship between severe maternal morbidity and post-traumatic stress disorder, which justified the combination of experiencing a life-threatening complication and its management, which may culminate in psychological trauma (61).

Lastly, the study identified postpartum intimate partner violence to be a significantly associated factor. Postpartum women who faced intimate partner violence during the postpartum period were more likely to develop PPTSD. This is supported by a study conducted in Brazil (23). This might be due to the fact that mothers who experienced intimate partner violence during the postpartum period may be more susceptible to feelings of isolation and hopelessness, which may lead to the development of PPTSD and other mental disorders.

## Strength and limitation

This study was one of the few studies conducted in Ethiopia regarding this topic, specifically among this population category. In addition, it was a community-based study, so it helped address all postnatal women. On the other hand, since this study was conducted with a cross-sectional study design, it does not show a cause-and-effect relationship among factors.

## Conclusion

Based on this study, it was identified that one out of five postnatal mothers suffers from PPPTSD. Parity, ANC follow-up, mode delivery, instrumental delivery, maternal morbidity, and postpartum intimate partner violence were found to be factors significantly associated with PPPTSD.

## Recommendation

We recommend that health professionals and health extension workers (HEWs) strengthen their routine activities by providing advice and educating pregnant women on the importance of ANC follow-up. In addition to this, health professionals should be strengthened to screen for common maternal morbidity complications during maternity visits and work on them.

It is recommended that clinicians spend a lot of time advising/counseling before and after procedures like cesarean birth and instrumental delivery, as it enhances the psychological preparation of mothers. Clinicians should also give more attention to ignored common mental disorders like PPTSD as, if it is not detected early and managed accordingly, these lead to devastating impacts for mothers, newborns, and families. In order to address the issue of intimate partner violence, health extension workers should encourage the mothers’ partners to participate in maternity and child services. In addition, they should screen for mothers who have experienced intimate partner violence and offer them psychological support.

The West Arsi Zonal Health Office, in collaboration with relevant organizations, ought to direct their attention toward this neglected issue and provide PPTSD training for healthcare professionals, as these can bridge knowledge gaps and enhance their focus on this topic. For researchers, it is better if studies are supported by strong study designs. Last but not the least, the existence of some postpartum mental health problems can affect the development of others. Thus, we recommend that the next researchers investigate the association between PPTSD and other common postpartum mental disorders.

## Data Availability

The raw data supporting the conclusions of this article will be made available by the authors, without undue reservation.
